# Three-Dimensional Printed Lingual Arch Space Maintainer: A Game Changer in Pediatric Dentistry

**DOI:** 10.7759/cureus.63680

**Published:** 2024-07-02

**Authors:** Ashish V Trivedi, Rajesh Aduri, Rehan Khan, Meenal S Pande

**Affiliations:** 1 Department of Pediatric Dentistry, Saraswati-Dhanwantari Dental College and Hospital, Parbhani, IND; 2 Department of Pediatric and Preventive Dentistry, Saraswati-Dhanwantari Dental College and Hospital, Parbhani, IND; 3 Department of Pediatric and Preventive Dentistry, Sharad Pawar Dental College, Datta Meghe Institute of Higher Education and Research (DU), Wardha, IND

**Keywords:** space loss, space maintainers, preventive orthodontics, three-dimensional printing, lingual arch, digital dentistry

## Abstract

Early loss of deciduous teeth is a challenging situation to handle. In recent years, the loss of deciduous teeth has become very frequent because of the increased risk of caries. Space maintainers play a vital role in preventing space loss. Lingual arch space maintainers are effectively used to maintain space in the lower arch. In order to retain the length of the lower arch and to prevent mesial migration of the mandibular first permanent molar, lingual arch space maintainers are often indicated. Conventional lingual arch fabrication is technique-sensitive and cumbersome. Additionally, it has many documented drawbacks like solder breakage, cement loss, soft tissue lesions, etc. With the advent of newer technology like three-dimensional (3D) printing, the fabrication of appliances and prostheses has become more predictable, accurate, and relatively easier. The present case report highlights the technique and advantages of 3D printing to fabricate lingual arch space maintainers, which has the potential to revolutionize preventive orthodontics in pediatric dentistry.

## Introduction

The function of primary teeth is to maintain inborn space until permanent teeth erupt, in addition to aiding in speech and mastication [1]. The emphasis on the primary dentition's role as the best space maintainer for permanent teeth is appropriate. Space maintainers are the recommended orthodontic appliance for the prevention of space loss because they retain space within the oral cavity [2]. It is best to use a space maintainer as soon as possible after early tooth loss because the greatest space loss is seen within a month of losing the deciduous teeth [3].

Until the permanent tooth erupts, the space maintainer maintains the spaces caused by the early loss of deciduous teeth. Conventional space maintainers have serious drawbacks despite their clinical effectiveness. These include the need for patient compliance, multiple steps in the fabrication process, extended chair-side times, soft tissue injury, technique-sensitive impressions and lab procedures, solder failure, caries formation at band margins, and long appliance fabrication times [4].

These flaws in the conventional space maintainer suggest the need for more advanced appliance designs and materials, one of which is three-dimensional (3D) printing, also commonly referred to as additive production or rapid prototype making [5]. The process involves creating 3D objects using a digitally scanned and layered design of the entire object. The design is preserved using a "Standard Tessellation Language" (STL) file. This case study describes how 3D printing technology was used to fabricate lingual arch space maintainers.

## Case presentation

**Figure 1:**
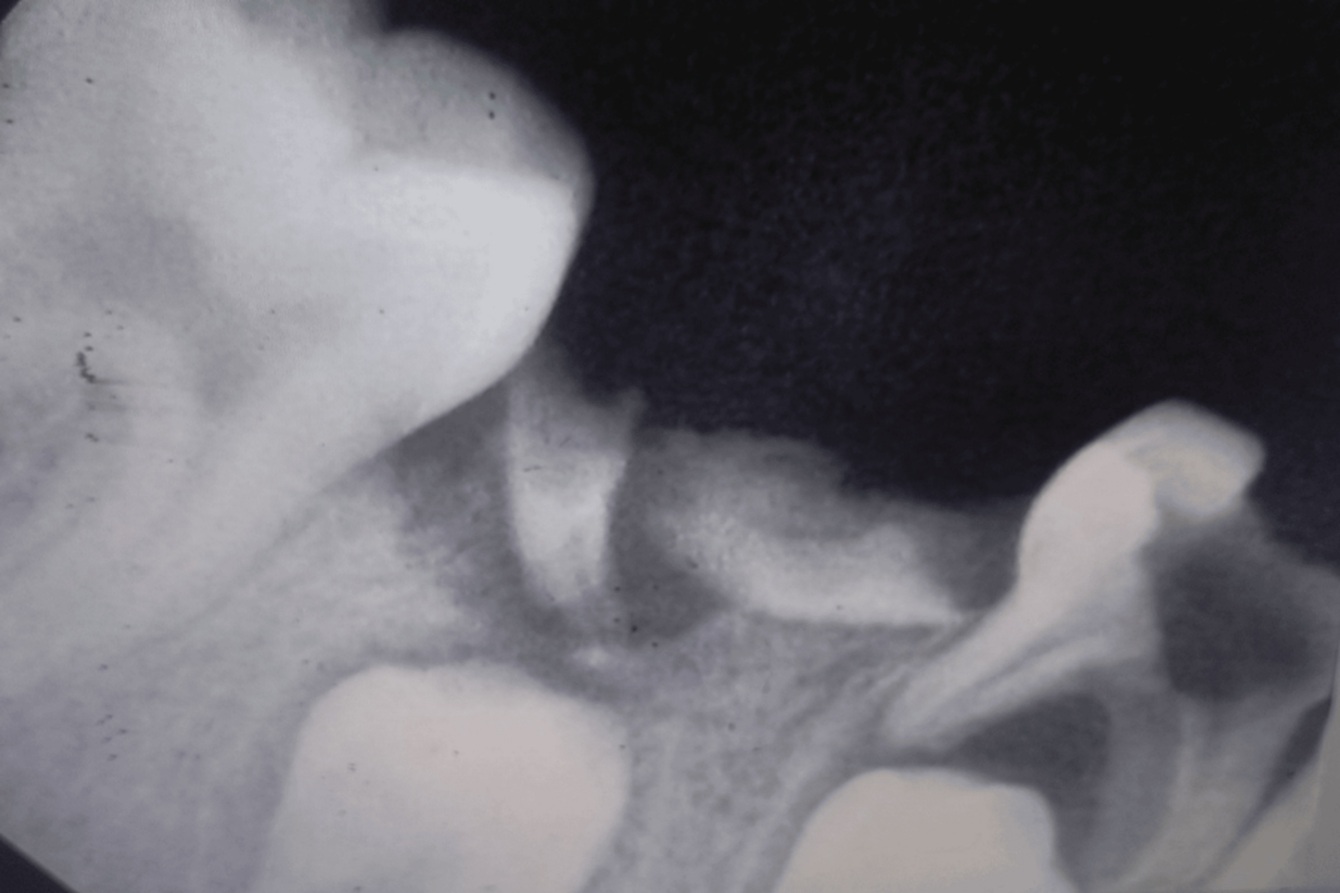
Intra-oral periapical radiograph of the mandibular left posterior region showing grossly carious 74, 75

**Figure 2:**
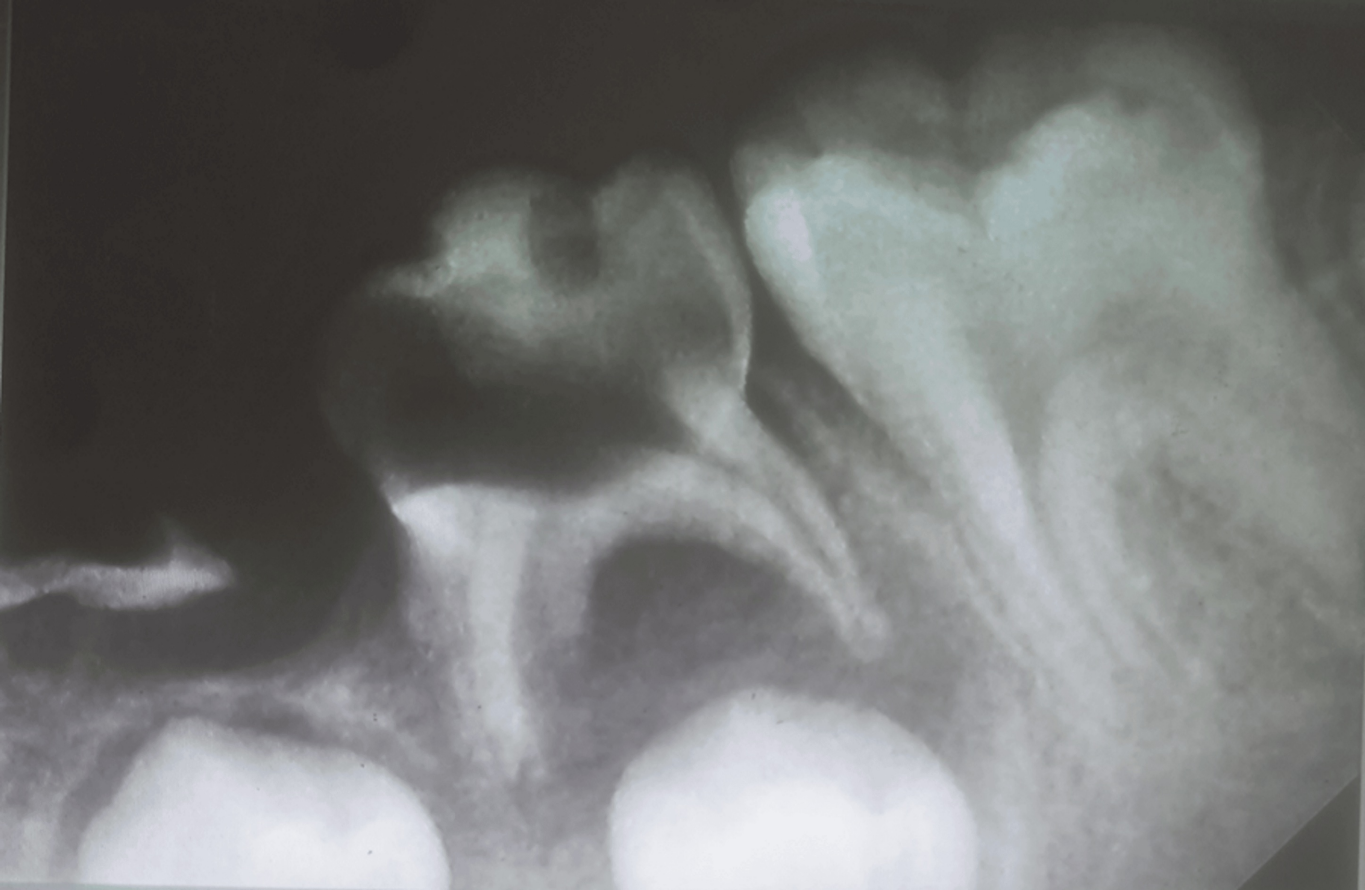
Intra-oral periapical radiograph of the mandibular right posterior region showing root stumps with 84 and complete fracture of mesial and lingual walls of 85

An eight-year-old boy presented with a chief complaint that he had been having trouble eating since last week from the right and left back tooth regions of his lower jaw. Clinical and radiographic examination of the mandibular arch showed grossly carious teeth in relation to 74 (Figure 1) and 84 (Figure 2). 75 showed furcal involvement and 85 showed a fracture of complete lingual and mesial walls which made them non-restorable and were indicated for extraction.

It was determined to develop a lingual arch space maintainer in order to prevent the space loss that would have occurred after extraction. It was done using the most latest 3D printing technology [5]. After explaining the process to the parent, written consent was obtained. Using additional silicone, a single visit putty impression was produced.

The extraction of all four carious teeth was done under infiltration with 2% lignocaine with 1: 1,00,0000 Adrenaline.

After extraction, the impression was sent to the three-dimensional printing lab to be scanned and produced using titanium-based powdered metal material (Ti64 Gd23; LPW Technology Ltd., Cheshire, UK) using Micro Laser Sintering Technology, which offers all the benefits of the additive manufacturing process.

Design and fabrication procedure

**Figure 3:**
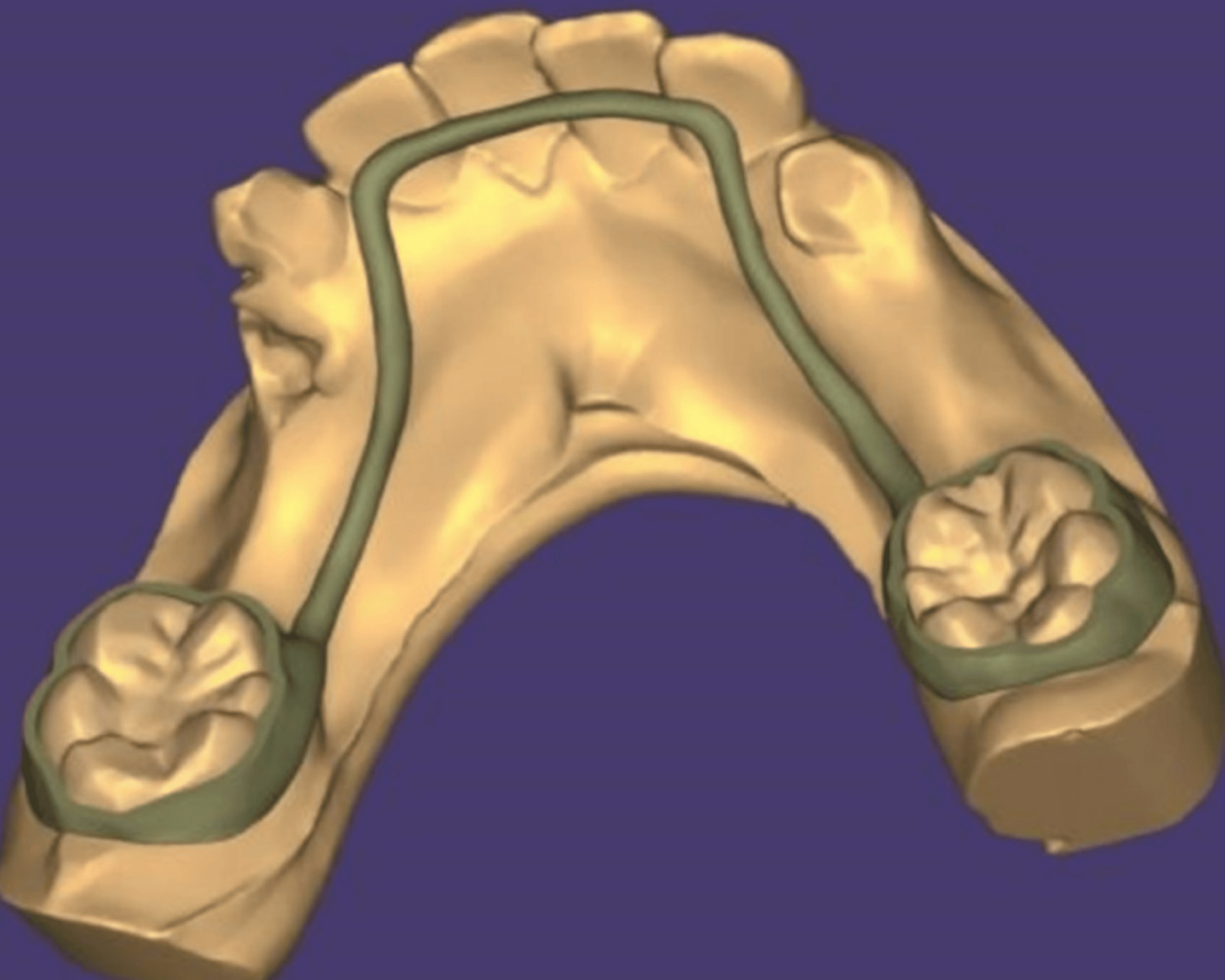
Desigining of the 3D lingual arch space maintainer

For scanning the cast, a three-dimensional digital dental scanner (Medit T500, Medit Corp., Seongbukgu, South Korea) was employed then after that, using DentalCAD 2.2 Valletta, the lingual arch space maintainer was fabricated to mimic the conventional space maintainer (Exocad GmbH, Darmstadt, Germany) (Figure 3). The three-dimensional space maintainer was printed deploying Micro Laser Sintering Technology, which provides all the features of an additive manufacturing technique, using a titanium-based powdered metal material (Ti64 Gd23; LPW Technology Ltd., Cheshire, UK).

**Figure 4:**
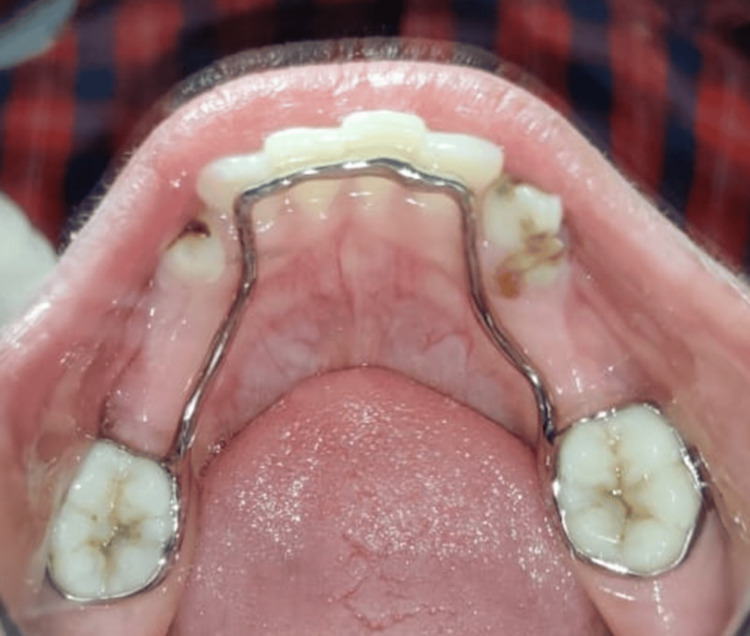
Post-operative occlusal view of the mandible after the insertion of the 3D printed lingual space maintainer

**Figure 5:**
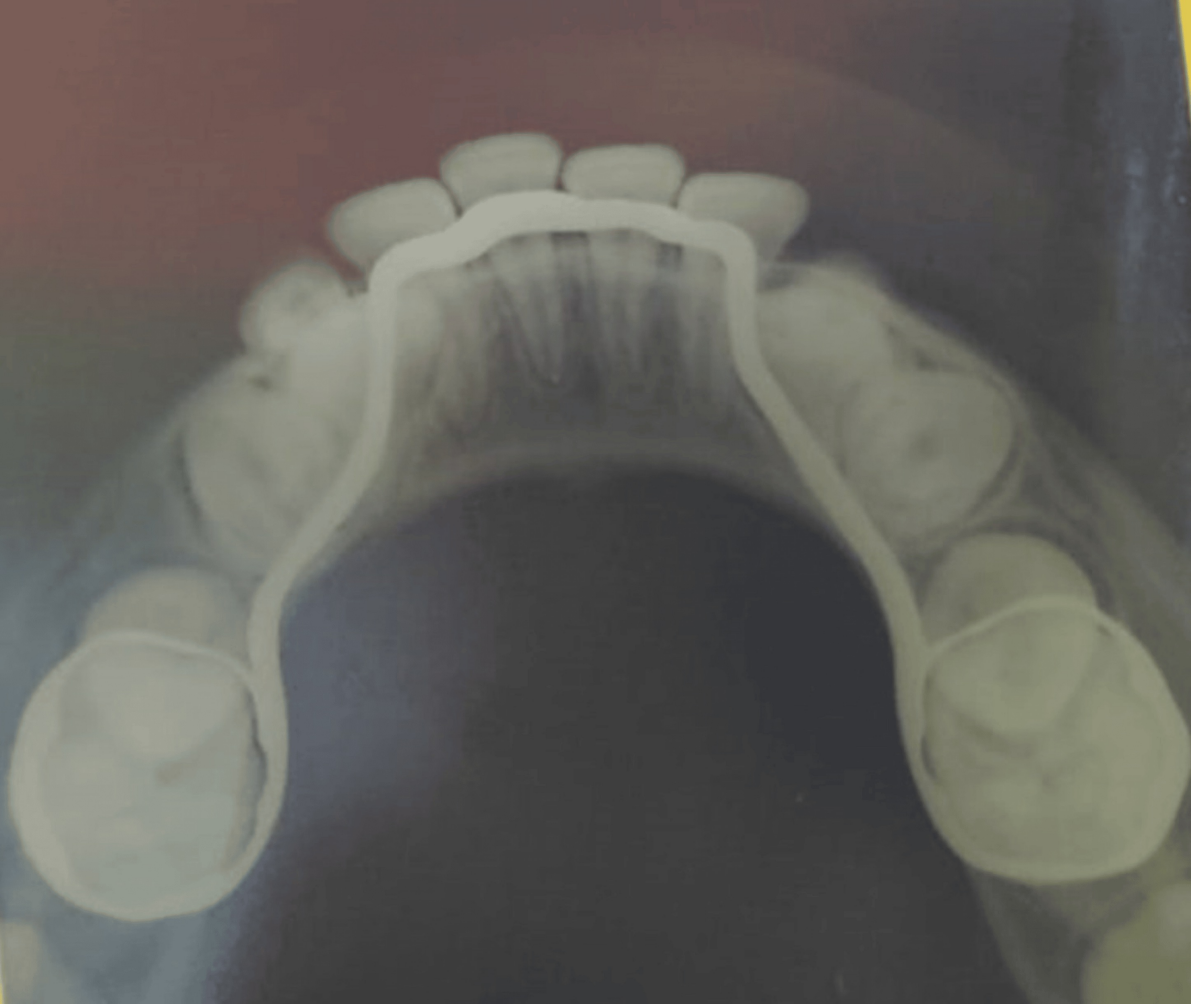
Radiographic occlusal view of the mandible showing the 3D printed lingual arch space maintainer

Subsequently, the printed space maintainer was evaluated in the patient's mouth to check for occlusion interferences, rocking, adaptation, and the gap between the tooth and band. In addition, luting glass ionomer cement (Type 1, Wizdent) was used to cement the lingual arch space maintainer that was 3D printed (Figures 4, 5).

## Discussion

A lower lingual arch is typically recommended as an effective space maintainer in order to maintain lower arch length, minimize mesial movement of the first permanent molar, lingual tilting of the incisors, and prevent the arch from imploding [6]. The early loss of primary molars, which results in 2-4 mm of gap closure per quadrant in both arches, has a significant effect on dental arch length. The mesial migration of the first permanent molar has been connected to the maximum amount of space loss. The benefit of a lingual arch has been utilized to keep the lower incisors in position and the molar from moving mesially [7].

There have been a few problems identified throughout the conventional space maintainers' construction that might lead it to fail. One of the main causes, according to Özüdoğru et al. [8], is solder breakage. As previously stated, the most frequent causes of appliance failure are solder breakage or cement loss [9]. The occlusal forces acting on the solder joint may be one reason for the early reduction of the traditional lingual arch space maintainer. Band pinching and transforming the band on the impression is a laborious task that is eliminated in the current design, in addition to being challenging for children.

One of the numerous benefits of 3D printing is that it makes appliances possible to be manufactured as one piece, thus reducing the possibility that the appliance might break. This is also explained by the easy adaptation, which keeps the occlusion from being impeded even in situations with strong forces of occlusion (Figure 3). 3D printing technology lowers human error, and multiple laboratory procedures are completed sooner [10]. The groundbreaking technology of 3D printing has revolutionary potential in dentistry. Crowns produced with intraoral scanners are already being manufactured employing this technology [11].

## Conclusions

The 3D printed lingual arch space maintainer showed accurate details like proper band adaption, loop ensuing gingival crest, and proper fit. It cannot, however, be regarded as the most cost-effective choice. However, the fact that three-dimensional printing strives to be a more predictable and minimally invasive procedure validates the expense. Additionally, chair-side time and laborious processes are minimized. Hence, 3D printing appliance fabrication is definitely going to increase the accuracy and predictability of space maintainer appliances in future.
